# Functional Analysis of Rare *RAS* Variants of Unknown Significance

**DOI:** 10.1158/2767-9764.CRC-25-0188

**Published:** 2025-10-02

**Authors:** Soohwan Park, Masachika Ikegami, Rina Kitada, Kazuya Takamochi, Kenji Suzuki, Hiroyuki Mano, Shinji Kohsaka

**Affiliations:** 1 Division of Cellular Signaling, National Cancer Center Research Institute, Tokyo, Japan.; 2 Department of General Thoracic Surgery, Graduate School of Medicine, Juntendo University School of Medicine, Tokyo, Japan.; 3 Department of Musculoskeletal Oncology, Tokyo Metropolitan Cancer and Infectious Diseases Center Komagome Hospital, Tokyo, Japan.

## Abstract

The *RAS* gene is frequently mutated in human cancers. Whereas the functional significance of frequent mutations is well established, the significance of rare mutations remains unknown. This study aimed to comprehensively investigate the function of rare *RAS* variants and provide new insights about their clinical relevance. A total of 298 *K/N/HRAS* variants (169, 72, and 57 variants, respectively) reported in the COSMIC database v100 were introduced into 3T3 cells. Subsequently, the drug sensitivity of *KRAS* variants to BI-2865, a noncovalent pan-KRAS inhibitor, was evaluated using the mixed-all-nominated-in-one method. The 3T3 focus formation assay newly identified 35 *KRAS*, 10 *NRAS*, and 21 *HRAS* variants as transforming competent. The oncogenicity assessed in the present study was consistent with that reported in the database. The drug sensitivity assay identified 15 *KRAS* variants sensitive to BI-2865. BI-2865 treatment inhibited the RAS downstream signaling pathways and induced apoptosis in cells with the sensitive variants. The present study identified 66 new oncogenic *RAS* variants. The sensitivity of *KRAS* variants to BI-2865 varies by variant. Functional analysis provides clues for the treatment of patients with rare *RAS* variants.

**Significance:**

This study presents the first comprehensive functional analysis of 298 rare *RAS* variants, identifying 66 novel oncogenic mutations and 15 *KRAS* variants sensitive to the noncovalent pan-KRAS inhibitor BI-2865. The heterogeneity in drug responses among *KRAS* variants underscores the need for variant-specific therapeutic strategies. These findings provide a preclinical framework for guiding personalized treatment in RAS-driven cancers and a valuable resource for understanding the clinical relevance of rare *RAS* mutations.

## Introduction

In human cancers, the *RAS* proto-oncogene family is frequently mutated, with alterations being observed in up to 20% of all cancers (1). KRAS, NRAS, and HRAS are the three main isoforms affected in cancer; among them, *KRAS* is the most frequent gain-of-function mutation, occurring in up to 90% of pancreatic cancer, 50% of colorectal cancer, and 30% of lung adenocarcinoma (2–6).

*KRAS* genes encode small GTP-binding proteins that act as a switch for many cellular signaling functions (6–8). The balance between nucleotide hydrolysis and exchange determines active KRAS levels in the cells. When bound to GDP, KRAS is in an “off” state. However, during GDP to GTP exchange, usually in response to growth factors and guanine nucleotide exchange factors such as SOS1/SOS2, KRAS cycles to its activated “on” state (9, 10). In this form, KRAS activates effector pathways (e.g., MAPK and PI3K pathways) to promote cellular proliferation and survival. When GTP is hydrolyzed to GDP (a process catalyzed by GTPase-activating proteins), KRAS returns to the “off” state (11). Among oncogenic *KRAS* mutations, many G12 and G13 mutations only significantly impair GAP-mediated hydrolysis and only Q61 mutations reliably impair intrinsic GTP hydrolysis, leading to the sustained activation of effector pathways and driving tumor growth (12, 13).

*KRAS* mutations are one of the most thoroughly studied targets for cancer therapy (14–16). Allele-specific inhibitors, which covalently bind to and trap *KRAS* G12C in an inactive state (17–19), have demonstrated clinical benefits in patients with lung cancer (20–22). Recently, the development of KRAS G12D and pan-KRAS inhibitors has advanced, thereby broadening the spectrum of therapeutic targets (23).

Being the most common *KRAS* subvariant in non–small cell lung cancer, *KRAS* G12C became the first targetable mutation with two FDA-approved drugs—sotorasib and adagrasib. To confirm the efficacy of sotorasib and adagrasib, their phase 3 trial, a head-to-head comparison against docetaxel, was read out. These studies confirmed their superiority over docetaxel in its primary endpoint (20, 24). However, because monotherapy has not been as effective as expected, clinical trials are ongoing to investigate combination therapy with immune checkpoint inhibitors or platinum doublet (25–27). Also, more potent KRAS G12C inhibitors are under development, and their clinical trials are ongoing. Divarasib is a novel KRAS G12C inhibitor, and its phase 1 study confirmed an objective response rate (ORR) of 53%, comparing favorably with currently approved inhibitors (28). Olomorasib is another promising KRAS G12C inhibitor with an ORR of 39% in patients who have experienced prior KRAS G12C inhibitors (29). Although G12C is the most common *KRAS* subvariant, the majority of *KRAS* mutations are non-G12C, including G12D/V/R, G13X, etc. These subvariants can also emerge as a secondary resistance mechanism. Thus, to fully drug *KRAS* mutations, strategies are urgently needed to expand to non-G12C (30). ASP3082 is a protein degrader specifically targeting *KRAS* G12D and is in the most advanced stage of development among similar drugs (31). Phase 1 clinical trials are currently conducted for solid tumors harboring KRAS G12D. Early phase I pan-tumor data showed an ORR of 33.3% (32). RMC-6236 is called a KRAS MULTI inhibitor that has activity across all *KRAS* mutations. In a phase 1 study of *KRAS* G12X, RMC-6236 resulted in an ORR of 38% in previously treated patients (33). Clinical trials with BI-2865 are currently not yet ongoing, but in preclinical stage, BI-2493, which is a structural analog of BI-2865 that was optimized for *in vivo* administration, attenuated tumor growth in mice bearing *KRAS* G12C, G12D, G12V, and A146V mutant models (30).

Although the functional significance of frequent mutations (e.g., *KRAS* G12C) is well established, the significance of rare mutations remains unknown. Recently, BI-2865, a noncovalent pan-KRAS inhibitor capable of engaging in a broader range of *KRAS* alleles, was developed (30). Therefore, we evaluated the transforming potential of *RAS* mutations and drug sensitivity of *KRAS* mutations in a high-throughput manner using the mixed-all-nominated-in-one (MANO) method developed in our laboratory (34). This system may provide new insights into the function of *RAS* variants of unknown significance (VUS).

## Materials and Methods

### Cell lines

Human embryonic kidney 293T cells (RRID: CVCL_0063) and 3T3 mouse fibroblasts (RRID: CVCL_0594) were purchased from the ATCC. The cells were cultured in DMEM-F12 supplemented with 10% FBS, 2 mmol/L glutamine, and 1% penicillin/streptomycin (all from Thermo Fisher Scientific). For all experiments, cells were used within 10 passages after thawing. Cell lines were not tested for *Mycoplasma* contamination within 6 months.

### Establishment of a retroviral vector with random barcodes

The pcx6 vector was constructed by inserting random 10–base-pair (bp) DNA barcode sequences upstream of the start codon of the target genes in the pcx4 vector (35). The barcode sequences are listed in Supplementary Table S1. The full-length cDNA for a human *K/N/HRAS* gene was inserted into the pcx6 vector. In this study, 298 *K/N/HRAS* variants (169, 72, and 57 variants, respectively) reported in the COSMIC database v100 (RRID: SCR_002260; https://cancer.sanger.ac.uk/cosmic) or GENIE 16.0-public (RRID: SCR_009197; https://www.aacr.org/professionals/research/aacr-project-genie/aacr-project-genie-data/) were selected. Plasmids encoding *K/N/HRAS* gene variants were created by AZENTA and sequenced by Sanger sequencing to confirm the full *K/N/HRAS* cDNA sequence and the 10-bp bar codes specific to each clone. Three clones containing an individual barcode were constructed for the respective variants to obtain triplicate data for all assays.

### Retrovirus production and 3T3 cell infection

To produce recombinant retroviruses, the recombinant plasmids were transduced together with packaging plasmids (Takara Bio) into human embryonic kidney 293T cells. The 3T3 cells were infected with ecotropic recombinant retroviruses in 96-well plates using 4 μg/mL of Polybrene (Sigma-Aldrich) for 24 hours.

### Focus formation assay

For focus formation assay (FFA), 3T3 cells expressing various *K/N/HRAS* variants were cultured in DMEM-F12 supplemented with 5% bovine calf serum for 2 weeks. Subsequently, the cells were stained with Giemsa solution (36). The FFA score was used to measure each variant’s transforming activity. This score classified the focus formation potential into the following groups: score 1 (negative), no focus formation; score 2 (mild), partial focus formation of transformed cells; score 3 (moderate), diffusely transformed cells piled up in bundles; and score 4 (severe), round-shaped and anchorage-independent foci diffusely observed. Variants exhibiting transforming potential equivalent to that of the wild-type (WT) are defined as mildly transforming (score 2), whereas those showing markedly higher transforming potential than the WT were classified as oncogenic variants (score 3 or 4).

### Proliferation competition assay

3T3 cells expressing various *KRAS* variants (169 variants), each tagged with a unique 10-bp barcode sequence, were mixed and cultured for 8 days in DMEM/F12 medium supplemented with 1.5% FBS. Cells were collected on days 4 and 8. Cell proliferation rates were determined by barcode sequencing using the Illumina MiSeq system (RRID: SCR_016379) and Reagent Kit v2 (300 cycles, cat. # MS-102-2002) and quantified based on the barcode counts corresponding to each variant. For details of the sequencing procedure, refer to the section of the MANO method.

### Drug sensitivity assay using the MANO method *in vitro*

A schematic of the MANO method is shown in Supplementary Fig. S1. This method uses a retroviral vector that enables the stable integration of individual genes into the genome of assay cells, including 3T3 cells, along with 10-bp barcode sequences. For the drug sensitivity assay, 3T3 cells expressing the respective *K/N/HRAS* gene variants were cultured in DMEM-F12 with 1.5% FBS. Subsequently, the cultured 3T3 cells were mixed and treated with the indicated concentrations (0.1 nmol/L–10 μmol/L) of BI-2865 (Selleck Chemicals, cat. # E1474) for 5 days. After the assay, genomic DNA was extracted from the cell lysates using the QIAamp DNA Mini Kit (Qiagen, cat. # 51306) and amplified by PCR with primers, indices, and adapters using Illumina technology. The primer sequences are described in Supplementary Table S2. The PCR products were purified using AMPure beads (Beckman Coulter, cat. # A63882). The sequencing libraries were prepared using the NEBNext Q5 Hot Start HiFi PCR Master Mix (New England Biolabs, cat. # M0543L) in accordance with the manufacturer’s instructions. The library quality was examined using the Qubit 4 Fluorometer (Thermo Fisher Scientific, cat. # Q33226, RRID: SCR_018095) and the Agilent 4200 TapeStation System (Agilent Technologies, cat. # G2991BA, RRID: SCR_018435). The resulting libraries were sequenced on an Illumina MiSeq system using the Reagent Kit v2 (300 cycles), and 150-bp paired-end reads were generated. The sequencing primers loaded into the MiSeq cartridge are described in Supplementary Table S3. The barcode sequences were included in the sequencing results, and the number of each barcode per variant was quantitated. Considering that the 3T3 cell variant had different doubling times, the cell viability of *HRAS* G12V at each concentration (0.1 nmol/L–10 μmol/L) in the PrestoBlue cell viability assay was used as the reference to normalize each clone’s barcode count. Each variant’s relative growth inhibition was calculated as the ratio of the average read number of triplicates to that of the control. Variants with less than 50 barcode sequence reads were excluded from the analysis. The assay was performed in triplicate.

### PrestoBlue cell viability assay

The transformed 3T3 cells expressing three *KRAS* gene variants (G12A, G12R, and Q61P) and *HRAS* G12V as a control variant were cultured in 96-well plates containing DMEM-F12 (100 μL of culture medium per well) with 1.5% FBS and BI-2865 at concentrations of 0.1 nmol/L to 10 μmol/L for 5 days. Subsequently, the plates were added with 10 μL of PrestoBlue (Thermo Fisher Scientific, cat. # A13261), and the fluorescence was measured after 30 minutes of incubation (excitation 530 nm, emission 590 nm) at 0.1 second. The fluorescence intensities of the wells without cells were used as negative controls. The data were analyzed and presented as a sigmoid curve using GraphPad Prism software v10 (RRID: SCR_002798). The IC_50_ value was defined as the inflection point on a dose–response curve.

### Western blot analysis

Parent 3T3 and 3T3 cells with individual variants were treated with the indicated BI-2865 concentrations for 1 day. Subsequently, the cells were lysed in 1% NP-40 lysis buffer containing protease and phosphatase inhibitors for 15 minutes on ice. Protein separation and detection were performed using an automated capillary electrophoresis system (Simple Western system and Compass software; ProteinSimple, RRID: SCR_025095). Antibodies against the following proteins were used: KRAS (1:10, E2M9G, cat. # 71835, RRID: AB_3705482), MEK1/2 (1:50, cat. # 9122, RRID: AB_823567), phospho-MEK1/2 (Ser217/221; 1:50, 41G9, cat. # 9154, RRID: AB_2138017), cleaved caspase-3 (Asp175; 1:50, 5A1E, cat. # 9664, RRID: AB_2070042), and GAPDH (1:50, 14C10, cat. # 2118, RRID: AB_561053). All primary antibodies were obtained from Cell Signaling Technology. Moreover, the signals were detected using a horseradish peroxidase–conjugated secondary anti-rabbit antibody and visualized using the ProteinSimple software.

## Results

### *RAS* mutations identified in human cancer

**Figure 1.**
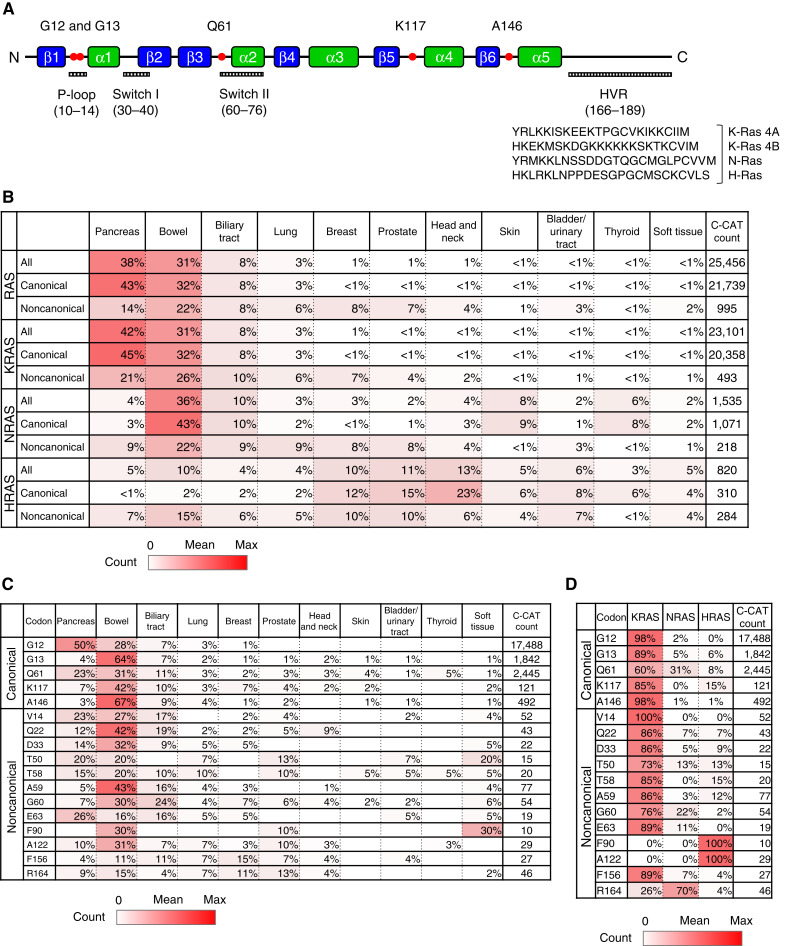
RAS protein domain and distribution of *RAS* mutations. **A,** RAS proteins comprise a G-domain consisting of five α-helix structures and six β-sheet structures, followed by a hypervariable region (HVR; 166–189 aa). The G-domain contains three GTP-binding domains and an effector-binding domain. The G-domain contains the P-loop (10–14 aa), switch I (30–40 aa), and Switch II (60–76 aa). **B,** Frequency of each cancer type associated with all mutations, canonical mutations, and noncanonical mutations in *RAS*, *KRAS*, *NRAS*, and *HRAS*, as observed in the C-CAT database. The values in each row represent percentages and sum to 100%, indicating the relative distribution of cancer types within each *RAS* mutation class. **C,** Frequency of each cancer type associated with codon-specific canonical and noncanonical *RAS* mutations, as observed in the C-CAT database. The values in each row represent percentages and sum to 100%, indicating the relative distribution of cancer types within each *RAS* mutation variant. **D,** Frequency of each RAS isoform among *RAS* mutations, as observed in the C-CAT database.

The GTP-binding domains comprise four regions: the phosphate-binding loop (P-loop, residues 10–17), switch I (residues 30–38), switch II (residues 60–76), and the base-binding loops (residues 116–120 and 145–147; Fig. 1A). Switch I and switch II are regions of interaction with downstream effectors (Fig. 1A). These regions regulate the nucleotide-dependent interactions between RAS proteins and their binding partners (37, 38). Most *KRAS* mutations occur in the G-domain (39).

We investigated the frequency of *RAS* mutations (*KRAS*, *NRAS*, *HRAS*) in different cancer types using the C-CAT database, a Japanese national cancer genome database. The frequency of *RAS* mutations in each cancer type was as follows: 80% in pancreatic cancer, 59% in bowel cancer, 29% in biliary tract cancer, 19% in ovarian/fallopian tube cancer, and 16% in lung cancer (Table 1). When stratified by isoform, *KRAS* mutations were observed in approximately 80% of pancreatic cancers and 54% of bowel cancers. *NRAS* mutations were frequently observed in skin and thyroid cancers. Lastly, *HRAS* mutations were generally rare but relatively frequent in bladder/urinary tract cancers and head and neck cancers. These findings were largely consistent with those reported in a previous study (40).

**Table 1.** Incidence of *RAS* mutations in cancer.

Cancer types	*KRAS*		*NRAS*		*HRAS*		Pan-RAS	
	*N*	%	*N*	%	*N*	%	*N*	%
Pancreas	9,670	79%	56	<1%	42	<1%	9,768	80%
Bowel	7,207	54%	553	4%	79	<1%	7,839	59%
Biliary tract	1,826	26%	149	2%	32	<1%	2,007	29%
Ovary/fallopian tube	755	17%	54	1%	26	<1%	835	19%
Lung	683	14%	49	1%	36	<1%	768	16%
Uterus	516	19%	59	2%	24	<1%	599	22%
Esophagus/stomach	488	10%	59	1%	29	<1%	576	12%
Other	394	19%	42	2%	22	1%	458	22%
Cervix	274	14%	18	<1%	15	<1%	307	15%
Breast	250	5%	47	<1%	82	2%	379	7%
Ampulla of Vater	244	51%	8	2%	2	<1%	254	54%
Prostate	143	3%	38	<1%	90	1%	271	6%
Bladder/urinary tract	119	9%	24	2%	50	4%	193	15%
Head and neck	84	3%	57	2%	110	4%	251	9%
Soft tissue	80	3%	32	1%	37	1%	149	5%
Central nervous system/brain	72	3%	33	1%	16	1%	121	5%
Skin	56	4%	118	9%	38	3%	212	16%
Testis	45	39%	4	4%	2	2%	51	45%
Peritoneum	43	8%	6	1%	5	<1%	54	10%
Liver	37	5%	12	2%	4	<1%	53	7%
Thyroid	29	3%	87	10%	23	3%	139	17%
Thymus	25	4%	9	1%	27	4%	61	10%
Bone	17	3%	4	<1%	3	<1%	24	4%
Vulva/vagina	16	9%	9	5%	8	4%	33	18%
Kidney	15	2%	0	<1%	5	<1%	20	3%
Adrenal gland	4	2%	1	<1%	3	1%	8	4%
Eye	4	4%	3	3%	3	3%	10	9%
Peripheral nervous system	4	1%	3	<1%	2	<1%	9	3%
Pleura	1	<1%	1	<1%	1	<1%	3	<1%
Penis	0	<1%	0	<1%	4	7%	4	7%
Total	23,101	29%	1,535	2%	820	1%	25,456	32%

The table shows the number of cases (*N*) and frequency (%) of mutations in *KRAS*, *NRAS*, and *HRAS* across various cancer types, as well as the combined total of all *RAS* isoform mutations (pan-RAS) according to the C-CAT database. The percentages represent the proportion of each primary tumor type that harbors a mutation in the specified *RAS* gene, relative to the total number of samples for that tissue type. The total number of RAS-mutant samples analyzed is indicated at the bottom of each column.

Additionally, tissue-specific mutations were identified in the C-CAT database (Supplementary Fig. S2; Supplementary Table S4). In lung cancer, *KRAS* G12C was the second most frequent mutation (18.6%), whereas in pancreatic cancer, G12C accounted for only 2.0% of all *KRAS* mutations. *KRAS* G13D was frequently observed in bowel cancer (14.2%) and cervical cancer (16.0%). *NRAS* Q61R and Q61K mutations were more frequently observed in skin and thyroid cancers than in other cancer types. *HRAS* Q61R mutations were frequently observed in prostate cancers and head and neck cancers. These results were consistent with a previous report (41).

Canonical *RAS* mutations present in the GTP-binding domain (G12, G13, Q61, K117, and A146 variants) account for the majority of *RAS* mutations, and more than half of cases with *RAS* mutations were pancreatic and colorectal cancers (Fig. 1B). In contrast, some noncanonical *RAS* mutations (T50, T58, G60, F90, F156, and R164 variants) were less frequent in pancreatic and colorectal cancers but more frequent in cancers of the biliary tract, lung, breast, prostate, and soft tissue (Fig. 1C). KRAS was the most frequently mutated isoform among noncanonical mutations, whereas certain mutations such as F90 and A122 were found to be specific to *HRAS* (Fig. 1D).

### Evaluation of the transforming potential of *RAS* variants by FFA

**Figure 2.**
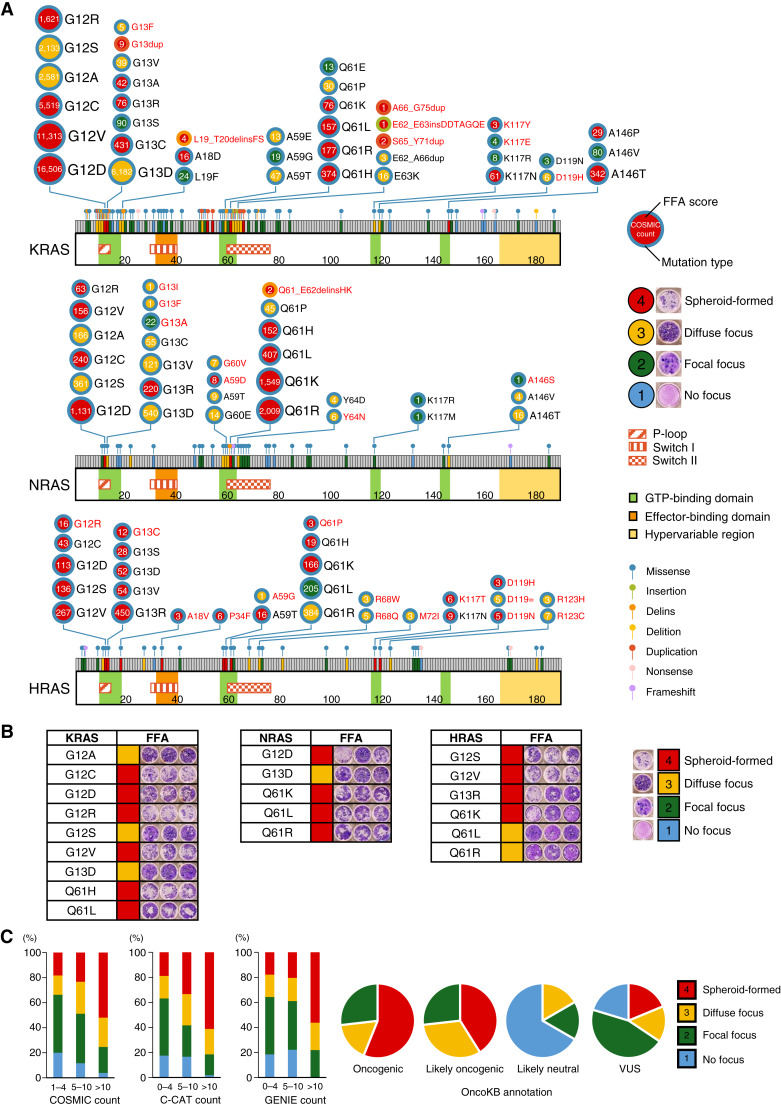
Lollipop chart and transforming activity of *RA*S variants. **A,** The results of the FFA for each RAS isoform are shown in the lollipop chart. The circle color indicates FFA scores, and the frame color of the circles indicates mutation type. The size of the circles was proportional to COSMIC count. **B,** The triplicate data of a representative variant in the FFA. **C,** Correlation of the FFA score of *RAS* variants with the variant count in the public databases or the OncoKB annotation. The oncogenicity evaluated as the FFA score is shown in colors and compared with the variant count number in the COSMIC, C-CAT, and GENIE databases. The distribution of FFA scores compared with OncoKB annotation is shown in the pie chart.

Although numerous studies have investigated the function of *RAS* genes (42, 43), most have focused on canonical mutations, such as those at codons G12, G13, and Q61. In this study, we conducted a comprehensive functional analysis of *RAS* genes. Specifically, we evaluated the transforming potential of 298 *K/N/HRAS* variants (169, 72, and 57 variants, respectively) reported in the COSMIC database using the 3T3 FFA. The results of the FFA for each RAS isoform are shown in the lollipop chart (Fig. 2A). The circle color indicates FFA scores, and the frame color of the circles indicates mutation type. The size of the circles was proportional to COSMIC count. The FFA results for *KRAS* (Fig. 2B; Supplementary Fig. S3A), *NRAS* (Fig. 2B; Supplementary Fig. S4A), and *HRAS* (Fig. 2B; Supplementary Fig. S5A) were evaluated along with COSMIC count and OncoKB annotations (Supplementary Table S5).

For *KRAS* variants, 43 variants (25.4%) had an FFA score of 4, 25 variants (14.8%) had an FFA score of 3, 73 variants (43.2%) had an FFA score of 2, and the remaining 28 variants (16.6%) had an FFA score of 1. All *KRAS* mutations affecting G12, G13, Q61, K117, and A146 had FFA scores of 2 to 4. All 14 G12 missense mutations reported in COSMIC exhibited strong transforming activity (FFA score of 3 or 4). Thirty-five variants were newly identified as transforming competent (defined as FFA scores of 3 or 4; Supplementary Fig. S3A). Those 35 variants were all rare variants (COSMIC count <10).

For *NRAS* variants, 15 variants (20.8%) had an FFA score of 4, 18 variants (25.0%) had an FFA score of 3, 25 variants (34.7%) had an FFA score of 2, and the remaining 14 variants (19.4%) had an FFA score of 1. Ten variants were newly identified as transforming competent (Supplementary Fig. S4A).

For *HRAS* variants, 24 variants (42.1%) had an FFA score of 4, 13 variants (22.8%) had an FFA score of 3, 17 variants (29.8%) had an FFA score of 2, and the remaining 3 variants (5.3%) had an FFA score of 1. Twenty-one variants were newly identified as transforming competent (Supplementary Fig. S5A).

The FFA score of *RAS* variants was compared with the variant counts in COSMIC, C-CAT, and GENIE. Most *RAS* variants with at least 10 cases reported in the database were transformed (96.1%, 98.1%, and 100.0% in COSMIC, C-CAT, and GENIE databases, respectively; Fig. 2C; Supplementary Figs. S3B, S4B, and S5B). All oncogenic or likely oncogenic *RAS* variants in OncoKB exhibited focus formation, whereas 66.7% of the variants annotated as likely neutral in OncoKB were not transformed (Fig. 2C; Supplementary Figs. S3C, S4C, and S5C). Among the 192 VUS in OncoKB, 66 variants (34.4%) had an FFA score of 3 or 4.

In the data presented in Hidalgo and colleagues (44), the proliferation rates of *HRAS* and *KRAS* missense mutations were quantified as scores relative to the WT variant. Among the 66 new oncogenic *RAS* variants in our study, 31 variants were *HRAS* or *KRAS* missense variants. Of these 31 variants, 22 variants were identified as gain-of-function in the data presented in Hidalgo and colleagues, whereas the remaining nine variants showed proliferation rates comparable with the WT. None of the new oncogenic variants in our study were identified as loss-of-function in the data presented in Hidalgo and colleagues.

### Assessment of agreement between FFA scores for *KRAS* and non-*KRAS* variants

To evaluate the concordance between FFA scores assigned to *KRAS* variants and those assigned to non-*KRAS* variants, we constructed a confusion matrix and calculated standard classification performance metrics (Supplementary Fig. S6). The overall classification accuracy was 59.4% (95% confidence interval, 46.9%–71.1%), which was significantly higher than the no-information rate of 39.1% (*P* = 0.00052).

Next, we calculated Cohen’s κ statistic to quantify inter-score agreement. The unweighted and quadratic-weighted κ values were 0.416 and 0.651, respectively, indicating moderate to substantial agreement according to the criteria of Landis and Koch. These findings suggest that the model effectively captures ordinal relationships among scores and approximates true FFA scores with reasonable fidelity.

Sensitivity estimates for individual scores ranged from 36.8% (score 2) to 81.5% (score 4), whereas specificity ranged from 71.4% to 98.4%. Balanced accuracy was the highest for score 4 (76.5%) and lowest for score 2 (64.4%), reflecting variable discriminative performance across score categories. Positive predictive values ranged from 42.1% to 80.0%, indicating varying levels of classification confidence depending on the score.

### The proliferative capacity of the *KRAS* rare variants

In the FFA, we identified 35 *KRAS* variants as newly exhibiting oncogenic potential. However, these variants are extremely rare and infrequently reported, and it remains unclear whether they confer any evolutionary advantage for cancer. To address this question, we performed a co-culture assay in which these rare variants were mixed with various *KRAS* variants, including canonical mutations such as G12, G13 and Q61. The proliferative capacity of those variants was evaluated as the fold change in the growth rate relative to WT *KRAS* on day 4 and day 8.

Analysis based on the FFA score revealed that higher FFA scores were associated with greater proliferative capacity (Supplementary Fig. S7A). Variants with strong transforming activity (FFA score of 3 or 4) exhibited significantly higher proliferation compared with WT *KRAS* both on day 4 and day 8. These results indicate that the FFA score also correlates with proliferative capacity.

We next compared the proliferative capacity of the 35 rare *KRAS* variants in this study with that of other variants scoring 3 or 4 in the FFA (Supplementary Fig. S7B). No significant differences were observed between the rare variants and the major oncogenic variants. These results suggest that rare *KRAS* variants confer proliferative advantage to cancer cells equivalent to that of known oncogenic variants.

### Drug sensitivity screening against *KRAS* variants using the MANO method

**Figure 3.**
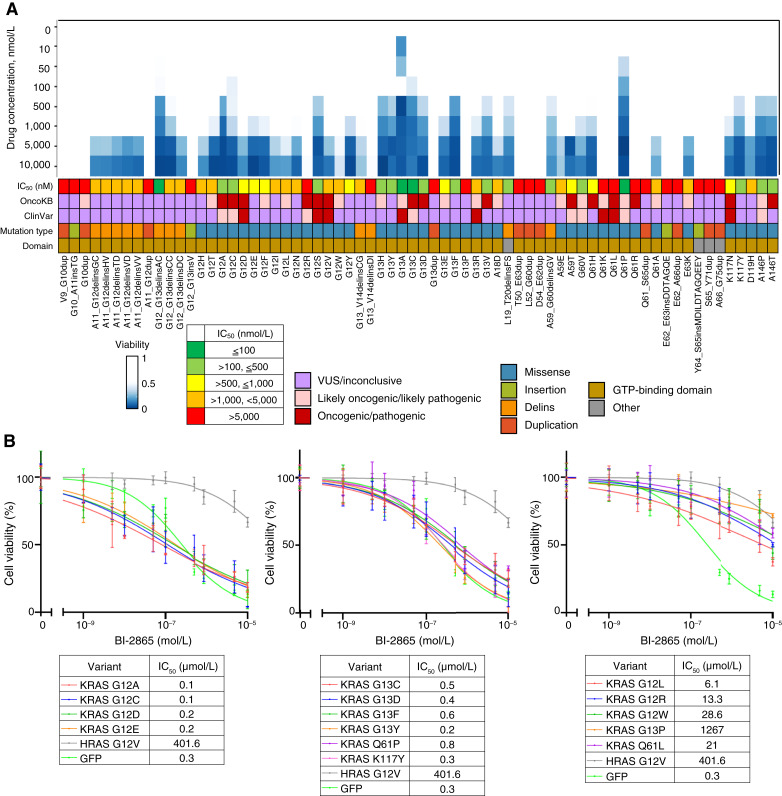
Drug sensitivity of *KRAS* variants to BI-2865. **A,** 3T3 cells expressing *KRAS* variants were treated with the indicated concentrations of BI-2865 (a pan-KRAS inhibitor). The viability of cells treated with BI-2865 was measured, and the results are illustrated using the color-coded scale. The IC_50_ values of BI-2865, the clinical significance annotated in the OncoKB and ClinVar databases, and the mutation type and domain are described at the bottom of the charts. **B,** 3T3 cells with *KRAS* variants, *HRAS* G12V, or GFP were incubated with the indicated concentrations of BI-2865 for 5 days. The cell viability was measured using the PrestoBlue cell viability assay and plotted relative to that of the untreated control. Data are presented as the mean ± SD (*n* = 6). The IC_50_ values of inhibitors are shown in the table on the right.

The drug sensitivity of *KRAS* variants was assessed using the MANO method. The mixture of 3T3 cells expressing individual *KRAS* variants was treated with different BI-2865 concentrations, as described in the “Materials and Methods” section. The sensitivity to BI-2865 was evaluated based on cell viability using the MANO method (Supplementary Tables S6 and S7). The cell viability of each variant was calculated as the ratio of the average read number of triplicates to that of the control. The drug sensitivity data of 68 *KRAS* variants that were identified as likely oncogenic or oncogenic by the FFA are shown in Fig. 3A. We analyzed the drug sensitivity of 72 *NRAS* and 57 *HRAS* variants to BI-2865 using the MANO method. However, no significant effect was observed for any *NRAS* or *HRAS* variants.

*KRAS* G12A/C/D/F/S/V, G13C/D, Q61H, K117N, and A146T were sensitive to BI-2865 (IC_50_ ≤ 1 μmol/L), whereas G12R and Q61L/R/K were resistant to BI-2865 (IC_50_ > 5 μmol/L). These results were consistent with those of previous studies (30) and demonstrated the validity of the MANO method. Among the 53 variants whose drug sensitivity to BI-2865 was not investigated in the previous study, we identified 15 sensitive variants, 17 strongly resistant variants, and 21 moderately resistant variants to BI-2865. Among the 15 sensitive variants, 12 (80.0%) were missense mutations. In contrast, 15 (88.2%) among the 17 resistant variants were either duplication or insertion mutations (duplication, 11 variants; insertion, 4 variants). All duplication and insertion variants with FFA scores of 3 or 4 exhibited resistance to BI-2865. Notably, several of the newly identified sensitive variants—G12_G13delinsAC, G12E, G13F/Y, L19_T20delinsFS, A59_G60delinsGV, and K117Y—were classified as VUS in OncoKB and ClinVar and had fewer than five reported cases in the COSMIC database (Supplementary Table S5).

The results of the drug sensitivity assay were validated using the PrestoBlue cell viability assay. In the PrestoBlue cell viability assay, the G12A, G12C, G12D, G12E, G13C, G13D, G13F, G13Y, Q61P, and K117Y variants showed a tendency toward more sensitive to BI-2865 than the G12L, G12R, G12W, G13P, and Q61L variants, although the difference was not statistically significant (*P* = 0.08; Welch *t* test; Fig. 3B). In addition, a positive correlation was observed among IC_50_ values evaluated by the MANO method and PrestoBlue cell viability assay (*r* = 0.98, Pearson’s correlation coefficient; Supplementary Fig. S8).

The ability of BI-2865 to inhibit downstream signaling pathway was evaluated through Western blot analysis (Supplementary Fig. S9). MEK1/2 phosphorylation was inhibited at 500 nmol/L in G12A (sensitive variant), whereas it is inhibited around 10 μmol/L in G12R (resistant variant). Cleaved caspase-3, a hallmark of apoptosis, was observed in 3T3 cells with *KRAS* G12A at BI-2865 concentrations above 500 nmol/L (Supplementary Fig. S9A). In addition, we performed immunoblot analysis for a subset of *KRAS* rare variants, including those newly identified in this study (G12E, G13F, G13P, and G60V), those predicted oncogenic (A18D, Q22K, and D33E), and duplication/delins variants (G10dup and L19_T20delinsFS). Compared with parental 3T3 cells, all those variants showed significantly increased MEK1/2 phosphorylation, suggesting activation of downstream signaling pathways (Supplementary Fig. S9B).

## Discussion

To the best of our knowledge, this study is the first to perform a comprehensive functional analysis of rare *RAS* variants with low frequency. Previous studies have analyzed the functional significance of more frequent mutations at *RAS* hotspots (G12, G13, and Q61; refs. 45–47). However, the significance of rare mutations remains to be fully elucidated. In the present study, the transforming potential and drug sensitivity to BI-2865 of 298 *K/N/HRAS* variants (169, 72, and 57 variants, respectively) were evaluated. In accordance with a previous study, we optimized the FBS concentration in the culture medium to ensure that the growth of 3T3 cells depended on activated RAS signaling (48).

KRAS mutants’ cycle between the “on” and “off” states and depend on nucleotide exchange for their activation (18, 49, 50). BI-2865 binds to a hydrophobic pocket located between switch I and switch II of KRAS mutant proteins in the GDP-bound “off” state (39). Therefore, structural alterations in switch I and switch II may impair drug binding, potentially leading to resistance. According to a previous study, GTP hydrolysis activity (GTPase activity) may affect drug sensitivity (39). Indeed, mutations such as G12R and Q61L/R/K are known to markedly reduce GTPase activity (51–54). Taken together, these findings suggest that reduced GTPase activity and structural alterations at the drug-binding site are major mechanisms of resistance to BI-2865.

Among the 17 variants that were newly identified as BI-2865 resistant by the MANO method, only G13P is a missense mutation. A plausible resistance mechanism of the G13P mutation is reduced GTPase activity. Substitution of glycine—a small, flexible, and nonpolar residue—with proline, a rigid amino acid with a cyclic structure, likely imposes conformational constraints on the P-loop, which spans residues G10 to V14. This structural restriction may hinder the dynamic conformational changes required for efficient GTP hydrolysis. The P-loop plays a critical role in stabilizing the phosphate groups of GTP and is essential for GTPase activity (55). Consequently, the G13P mutation is thought to impair GTP hydrolysis, resulting in accumulation of the GTP-bound (active) form of KRAS and subsequent resistance to BI-2865.

The remaining 16 resistant variants were primarily classified as insertions, duplications, or deletion–insertion mutations located in or around critical structural regions such as the P-loop (G10–V14) and switch II (G60–E76). Mutations involving insertions or duplications within these key structural elements are likely to induce substantial conformational changes in the KRAS protein, thereby profoundly affecting its GTPase function and structural integrity. Mutations affecting the P-loop, such as V9_G10dup, G10dup, G10_A11insTG, A11_G12dup, G12_G13insV, G13dup, and G13_V14delinsDI, are likely to impair the flexible phosphate-binding architecture required for GTP hydrolysis. Its conformation is highly sensitive to residue size and polarity. These mutants are biased toward the constitutively active, GTP-bound state of KRAS, thereby diminishing the availability of the GDP-bound conformation targeted by BI-2865. Furthermore, local structural distortion likely interferes with the inhibitor binding pocket itself, physically blocking BI-2865 access. Additionally, insertions or duplications located in the switch II region, such as T50_E63dup, L52_G60dup, D54_E62dup, Q61_S65dup, E62_E63insDDTAGQE, E62_A66dup, Y64_S65insMDILDTAGQEEY, S65_Y71dup, and A66_G75dup, may directly perturb the structural integrity of the BI-2865–binding surface, resulting in impaired drug binding and reduced inhibitory efficacy. Mutations in this region, particularly large insertions and tandem duplications, cause severe structural distortion, almost certainly inhibiting GTP hydrolysis and reconfiguring the BI-2865–binding surface beyond recognition. Collectively, these results suggest a unified mechanism for BI-2865 resistance in *KRAS* insertion and duplication mutations, highlighting that maintaining the native structure of important structural motifs—such as the P-loop and switch II—for inhibitor susceptibility. Furthermore, they underscore the need for future therapeutic strategies that can accommodate the conformational plasticity and structural heterogeneity introduced by rare *KRAS* insertions and duplications.

Clinical validation is needed to confirm these preclinical data. There are currently no clinical trials of BI-2865 and its structural analog of BI-2493. However, BI 3706674, a related compound, is under clinical investigation for patients with gastroesophageal cancers carrying *KRAS* amplifications (NCT06056024).

This study has potential limitations that should be acknowledged. First, although FFA using 3T3 cells is a well-known method to assess one aspect of the transforming potential of an oncogene (36), the functions of *RAS* genes in different cell contexts remain to be assessed. Therefore, future studies using other cellular contexts will be important to determine whether the observed transforming activities and therapeutic vulnerabilities of rare *RAS* variants are cell type–specific or broadly applicable across diverse tissue types. Second, evaluating the transforming potential of *RAS* variants is complicated because retroviral transduction into cell lines results in elevated RAS protein compared with endogenous RAS expression. However, the overexpression of WT RAS confers no transforming activity in 3T3 cells. Third, the preclinical data obtained in this study are not validated by clinical data. Thus, the findings must be confirmed in large-scale clinical studies. Because the drug sensitivity of rare *RAS* variants has rarely been validated in clinical practice, validation in basket-type clinical trials is needed. Finally, this study did not take into account other gene mutations (e.g., *TP53* and *PIK3CA* mutations) that can co-occur in cancers harboring *KRAS* mutations and affect drug sensitivity (56–58).

In conclusion, the functional analysis of rare *RAS* variants was successfully performed using the MANO method. We identified the transforming potential of 66 rare *RAS* variants. Among them, 15 *KRAS* variants were identified for the first time to be sensitive to BI-2865. Considering that each *KRAS* mutation exhibits various sensitivities to BI-2865, it is important to select the optimal inhibitor for a patient with a particular *KRAS* variant. This preclinical screening assay could be valuable for identifying the appropriate drug for treating rare *RAS* variants. Our functional analysis of *RAS* mutations offers a fundamental database to develop personalized treatment for RAS-driven cancers and provides clues to treating patients with rare variants.

## Supplementary Material

Supplementary Figure S1 Schema of the assays for the variant assessment

Supplementary Figure S2 The frequency of oncogenic *RAS* mutations in cancer according to the C-CAT database

Supplementary Figure S3 Transforming activity of* KRAS* variants

Supplementary Figure S4 Transforming activity of *NRAS* variants

Supplementary Figure S5 Transforming activity of *NRAS* variants

Supplementary Figure S6 Concordance of FFA scores between *KRAS* and corresponding HRAS/NRAS variants

Supplementary Figure S7 Proliferative capacity of *KRAS* variants in the co-culture assay

Supplementary Figure S8 Concordance in the sensitivity to pan-KRAS inhibitor assessed through the MANO method and PrestoBlue cell viability assay

Supplementary Figure S9 Immunoblot analysis of *KRAS* variants in 3T3 cells treated with BI-2865

Supplementary Table S1 The barcode sequence of each variant clone

Supplementary Table S2 Primer sequences used for barcode amplification

Supplementary Table S3 Primer sequences used for next-generation sequencing

Supplementary Table S4 Prevalence of canonical *KRAS* variants at mutant allele level

Supplementary Table S5 Details of the 298 variants

Supplementary Table S6 Raw data for Figure 3A

Supplementary Table S7 Details of the drug sensitivity assay

## Data Availability

The data generated in this study are available within the article, its supplementary data files, and upon reasonable request from the corresponding author.
