# Supplementary material for: Functional Analysis of Rare RAS Variants of Unknown Significance
Source: Cancer Res Commun. 2025 Oct 2;5(10):1747–57. doi: 10.1158/2767-9764.CRC-25-0188 (PMC12488390; doi:10.1158/2767-9764.CRC-25-0188)
Supplement: Supplementary Figure S1 — Schema of the assays for the variant assessment [file crc-25-0188_supplementary_figure_s1_suppsf1.docx]

**
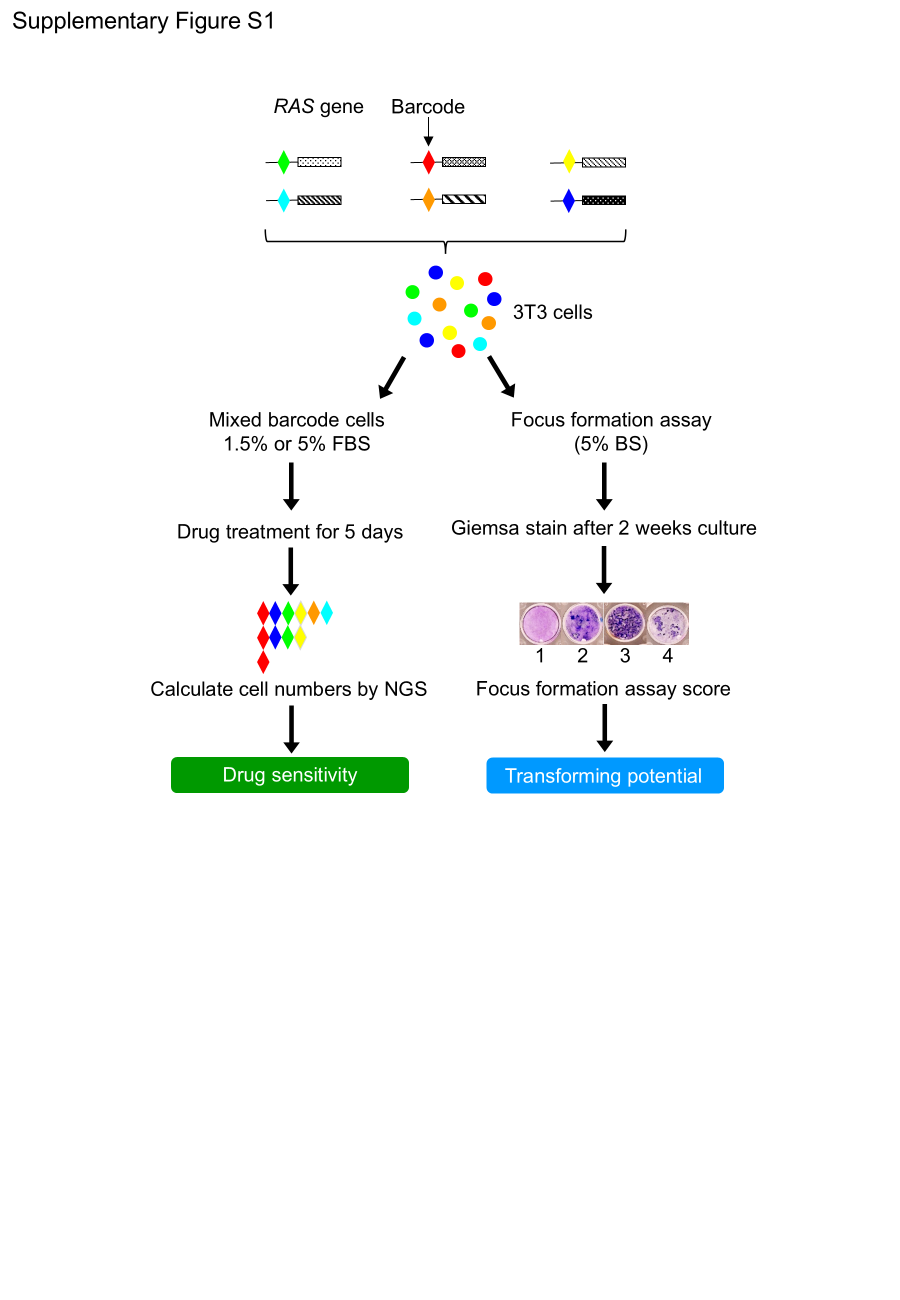
**

**Supplementary Figure S1. Schema of the assays for the variant assessment**

Retroviral vectors stably integrate individual genes with barcode sequences into the genome of 3T3. After variant introduction, the cells were collected and cultured in a pool for drug sensitivity assessment by the MANO method (left panel) or individually assessed for transforming potential (right panel). The details of the assay are provided in the Materials and Methods section.
