# Supplementary material for: Functional Analysis of Rare RAS Variants of Unknown Significance
Source: Cancer Res Commun. 2025 Oct 2;5(10):1747–57. doi: 10.1158/2767-9764.CRC-25-0188 (PMC12488390; doi:10.1158/2767-9764.CRC-25-0188)
Supplement: Supplementary Figure S2 — The frequency of oncogenic RAS mutations in cancer according to the C-CAT database [file crc-25-0188_supplementary_figure_s2_suppsf2.docx]

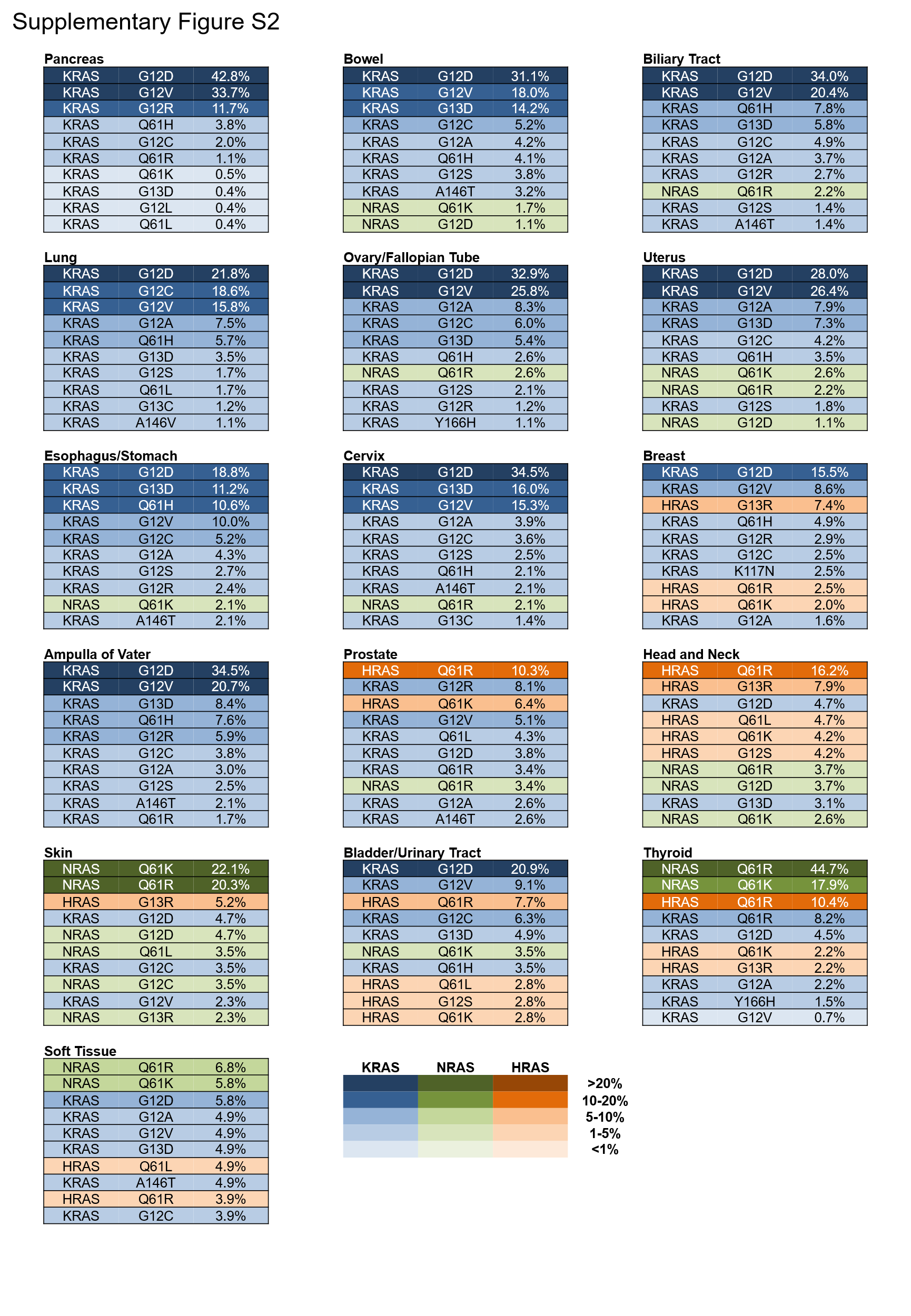


**Supplementary Figure S2. The frequency of oncogenic *RAS* mutations in cancer according to the C-CAT database**

Cancer types associated with *RAS* mutations (over 100 tumor samples) are displayed on the panel. For each tumor type, the top 10 most frequent substitutions in RAS isoforms and their frequencies are listed. The color of the mutation indicates the mutated *RAS* gene (*KRAS*, blue; *NRAS*, green; and *HRAS*, orange). The data was obtained from the C-CAT database.
