# Supplementary material for: Functional Analysis of Rare RAS Variants of Unknown Significance
Source: Cancer Res Commun. 2025 Oct 2;5(10):1747–57. doi: 10.1158/2767-9764.CRC-25-0188 (PMC12488390; doi:10.1158/2767-9764.CRC-25-0188)
Supplement: Supplementary Figure S4 — Transforming activity of NRAS variants [file crc-25-0188_supplementary_figure_s4_suppsf4.docx]

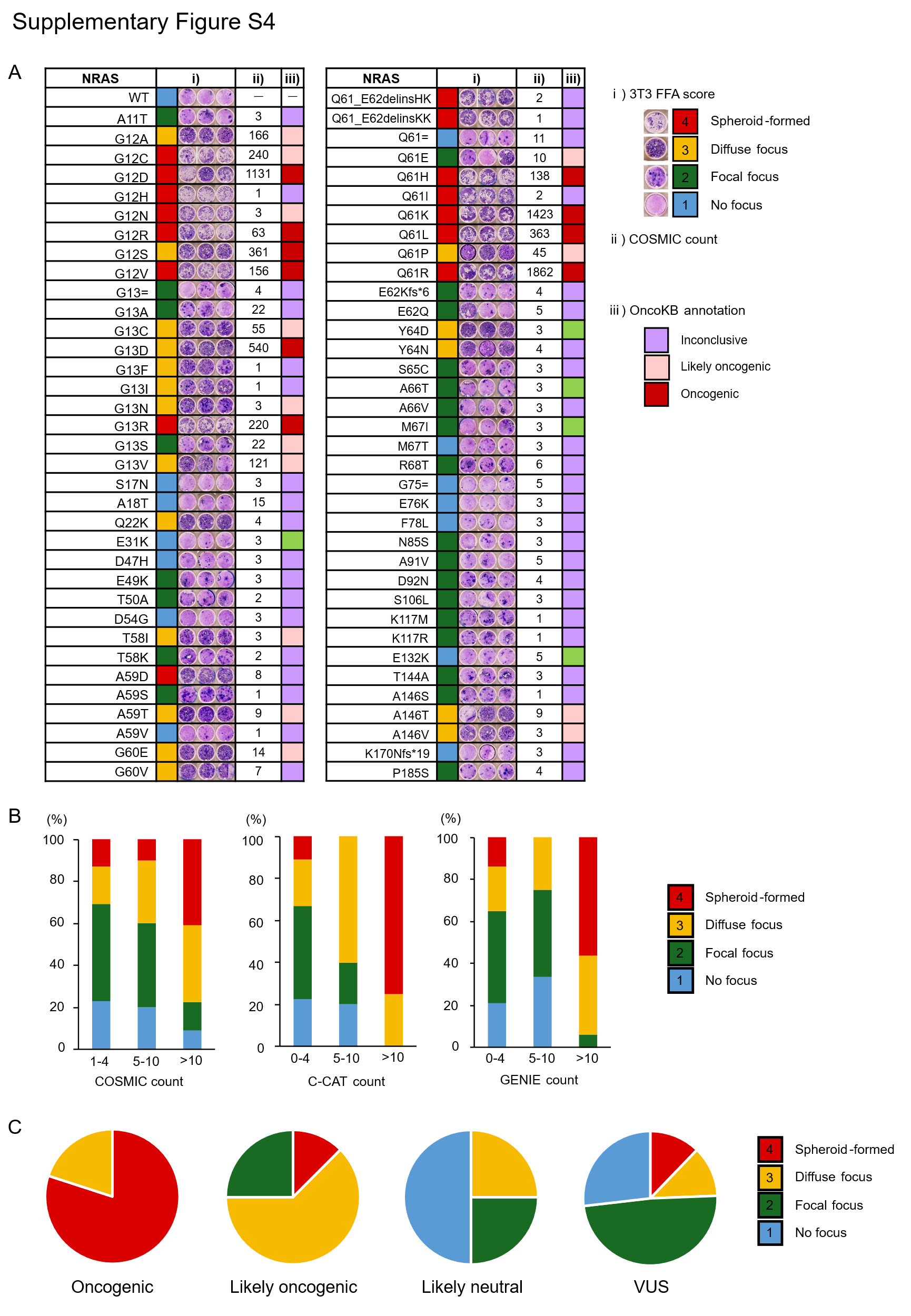


**Supplementary Figure S4. Transforming activity of *NRAS* variants**

(**A**) Information of *NRAS* variants. 3T3 The FFA scores (i), the number of COSMIC database v100 counts (ii), and the OncoKB annotation (iii) are indicated. (**B**) Correlation of the FFA score with the variant count in the public database. The oncogenicity evaluated as the FFA score is shown in colors and compared with the variant count number in the COSMIC, C-CAT, and GENIE databases. (**C**) Correlation of the FFA score with the OncoKB annotation. The oncogenicities evaluated as the FFA score are shown in colors and compared with those of OncoKB, which are shown at the bottom of the pie chart.
