# Supplementary material for: Functional Analysis of Rare RAS Variants of Unknown Significance
Source: Cancer Res Commun. 2025 Oct 2;5(10):1747–57. doi: 10.1158/2767-9764.CRC-25-0188 (PMC12488390; doi:10.1158/2767-9764.CRC-25-0188)
Supplement: Supplementary Figure S6 — Concordance of FFA scores between KRAS and corresponding HRAS/NRAS variants [file crc-25-0188_supplementary_figure_s6_suppsf6.docx]

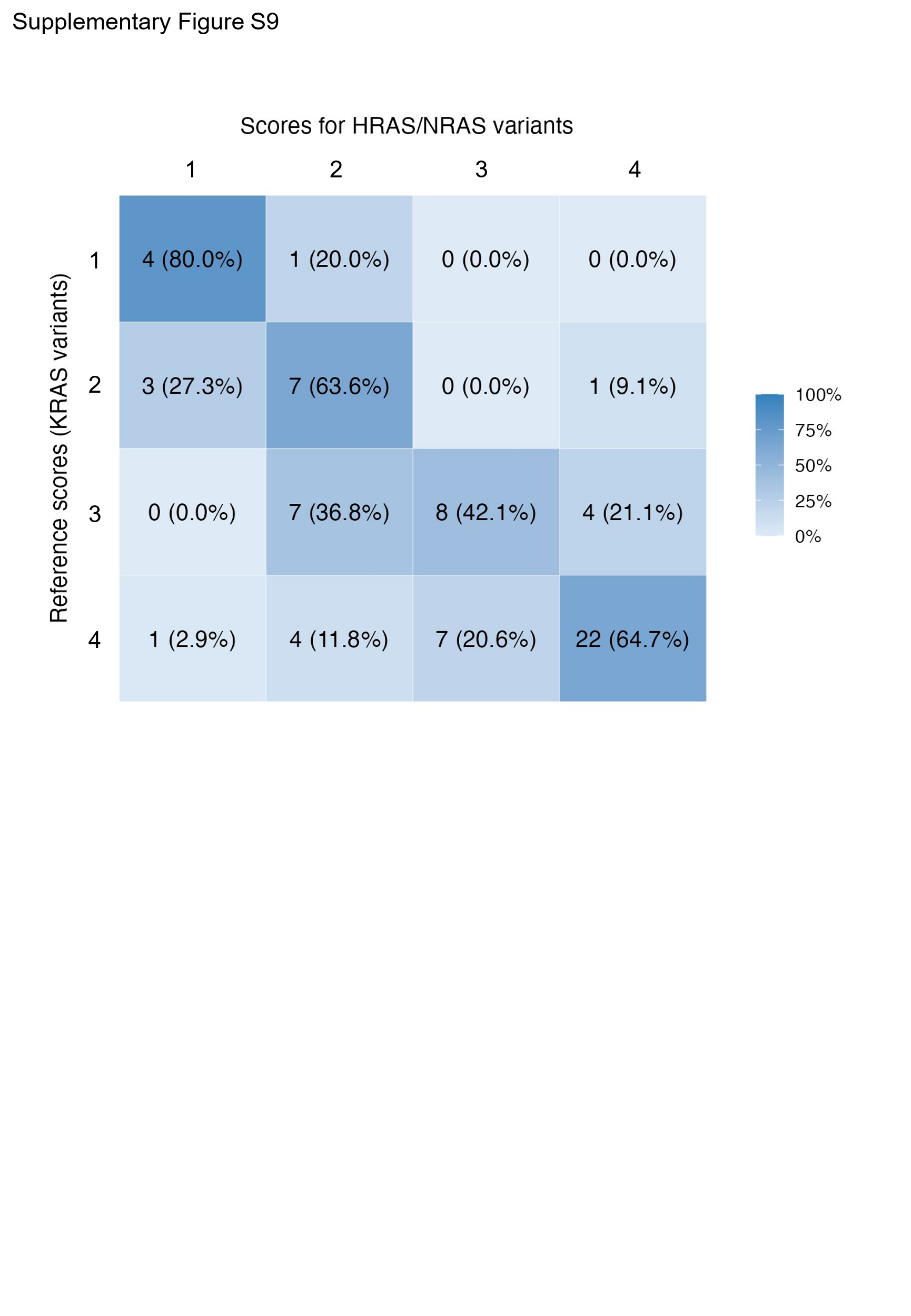


**Supplementary Figure S6. Concordance of FFA scores between *KRAS* and corresponding *HRAS/NRAS* variants**

A heatmap showing the concordance of FFA scores between *KRAS* variants (reference scores, y-axis) and the corresponding *HRAS* or *NRAS* variants (x-axis). Each cell represents the number and percentage of variants with the indicated score pairing. Higher percentages along the diagonal indicate a general consistency in transforming potential across RAS isoforms. The color intensity reflects the percentage within each row. Kappa statistics were computed using the irr package (version 0.84.1) in R.
