# Supplementary material for: Functional Analysis of Rare RAS Variants of Unknown Significance
Source: Cancer Res Commun. 2025 Oct 2;5(10):1747–57. doi: 10.1158/2767-9764.CRC-25-0188 (PMC12488390; doi:10.1158/2767-9764.CRC-25-0188)
Supplement: Supplementary Figure S7 — Proliferative capacity of KRAS variants in the co-culture assay [file crc-25-0188_supplementary_figure_s7_suppsf7.docx]

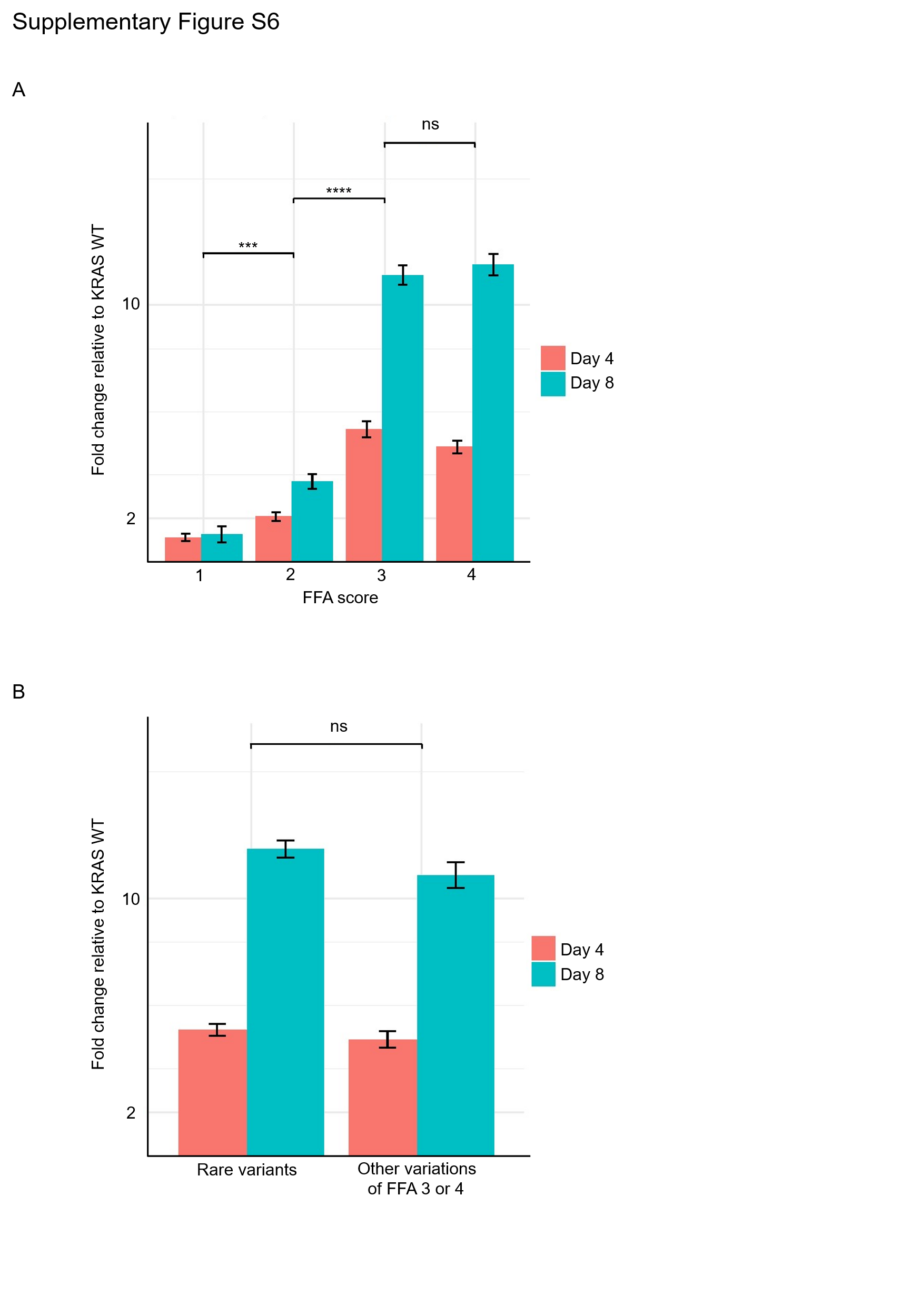


**Supplementary Figure S7. Proliferative capacity of *KRAS* variants in the co-culture assay**

**(A)** Relationship between FFA score and cell proliferation in the co-culture assay. *KRAS* variants were grouped by FFA score (1 to 4), and their proliferative capacity was measured on days 4 and 8, expressed as fold change relative to *KRAS* wild-type (WT). Higher FFA scores were associated with increased proliferation. Statistical significance was assessed by Wilcoxon test with Bonferroni adjustment (***p < 0.001, ****p < 0.0001, ns: not significant). **(B)** Comparison of proliferative capacity between rare *KRAS* variants identified in this study and other variants with FFA scores of 3 or 4. No significant difference in proliferation was observed between the two groups. Data are presented as mean ± standard deviation.
