# Supplementary material for: Functional Analysis of Rare RAS Variants of Unknown Significance
Source: Cancer Res Commun. 2025 Oct 2;5(10):1747–57. doi: 10.1158/2767-9764.CRC-25-0188 (PMC12488390; doi:10.1158/2767-9764.CRC-25-0188)
Supplement: Supplementary Figure S8 — Concordance in the sensitivity to pan-KRAS inhibitor assessed through the MANO method and PrestoBlue cell viability assay [file crc-25-0188_supplementary_figure_s8_suppsf8.docx]

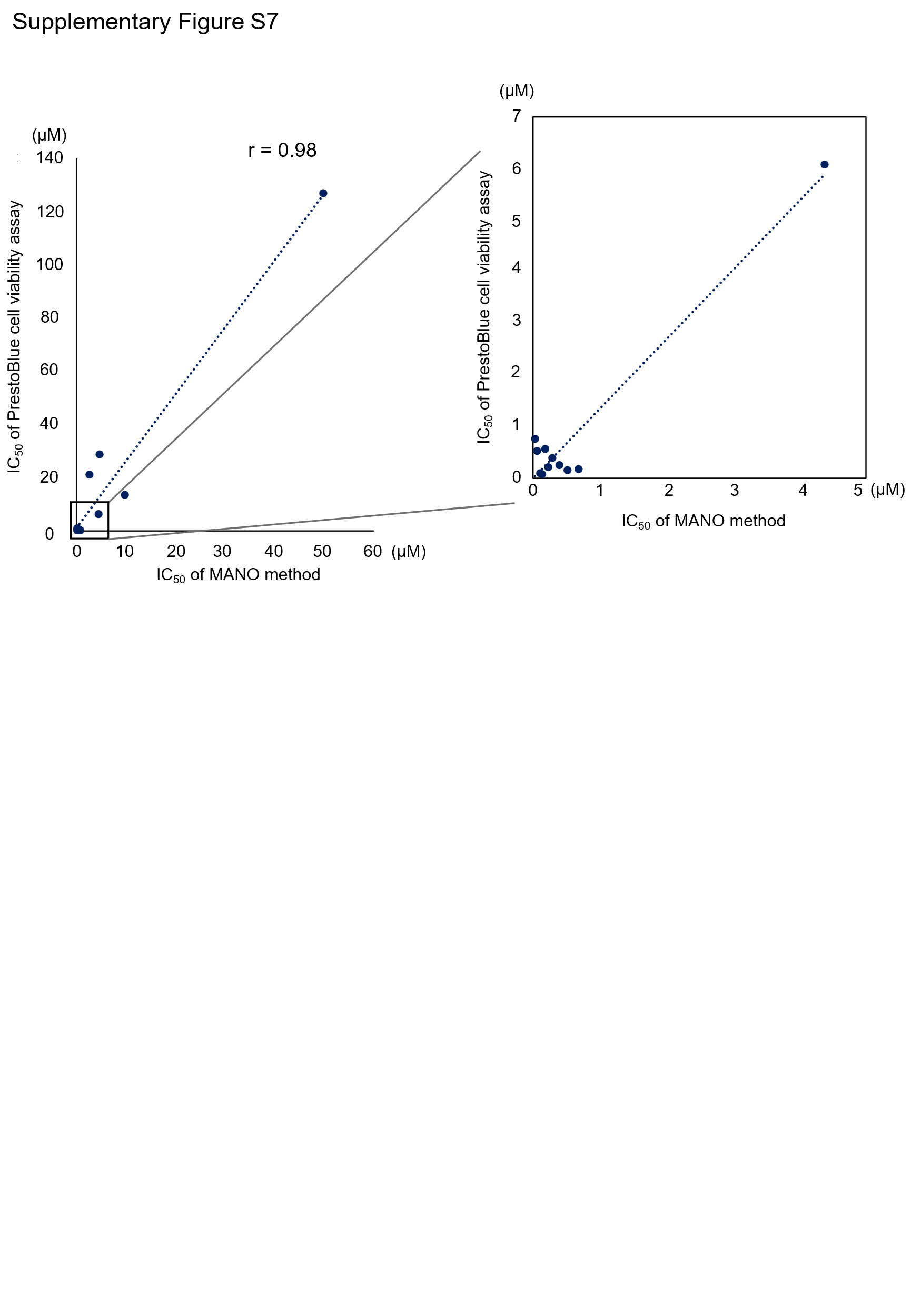


**Supplementary Figure S8. Concordance in the sensitivity to pan-KRAS inhibitor assessed through the MANO method and PrestoBlue cell viability assay**

Comparison of the PrestoBlue cell viability assay and the MANO method for the cell viability of 3T3 cells with 15 *KRAS* mutants shown in **Fig. 3B** after the treatment with pan-KRAS inhibitor. Each data point was normalized to data obtained from vehicle-treated cells. Pearson’s correlation coefficient (*r*) was 0.98 (*p* < 0.001). The low ratio area is magnified in the right panel.
