# Supplementary material for: Functional Analysis of Rare RAS Variants of Unknown Significance
Source: Cancer Res Commun. 2025 Oct 2;5(10):1747–57. doi: 10.1158/2767-9764.CRC-25-0188 (PMC12488390; doi:10.1158/2767-9764.CRC-25-0188)
Supplement: Supplementary Figure S9 — Immunoblot analysis of KRAS variants in 3T3 cells treated with BI-2865 [file crc-25-0188_supplementary_figure_s9_suppsf9.docx]

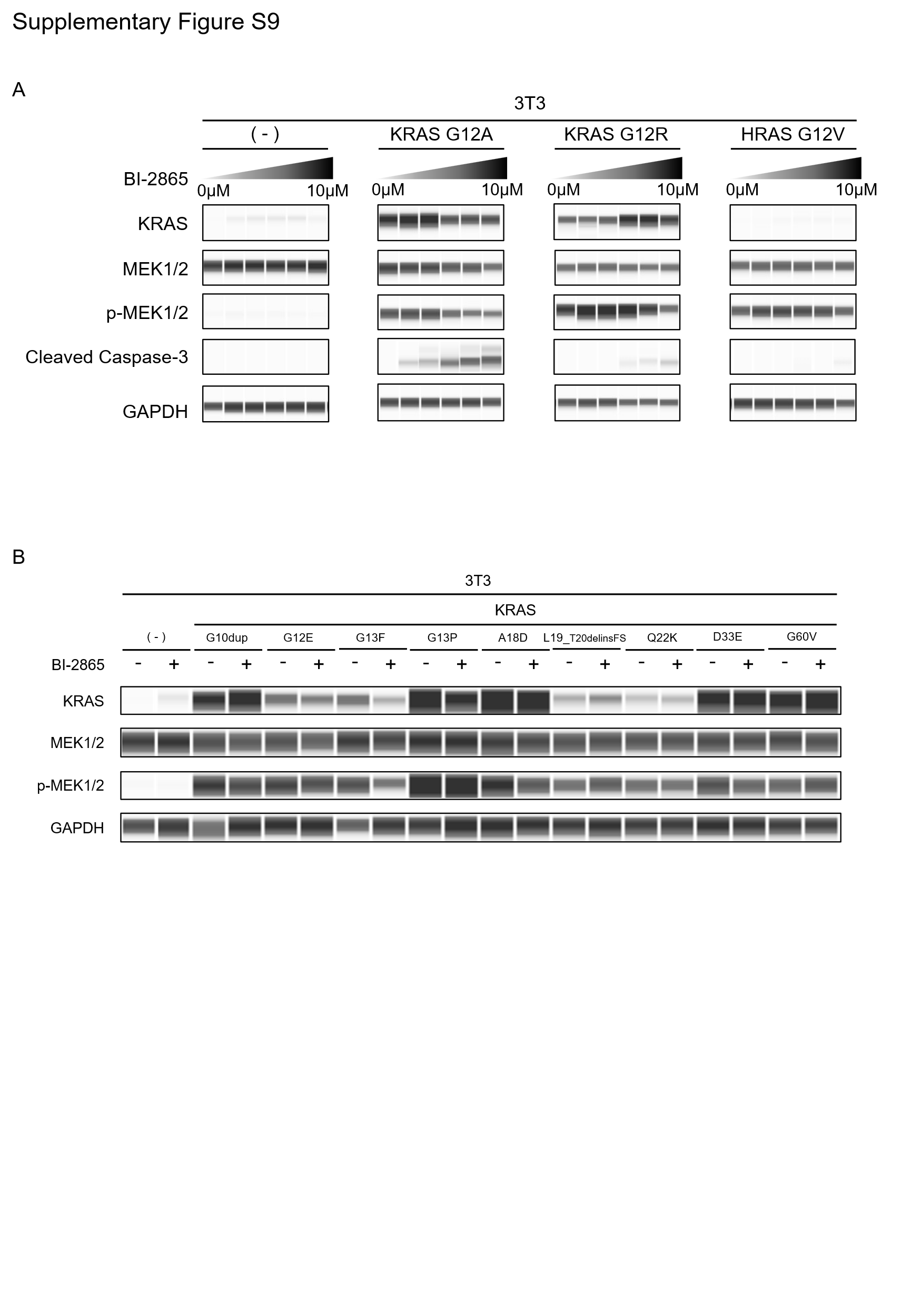


**Supplementary Figure S9. Immunoblot analysis of *KRAS* variants in 3T3 cells treated with BI-2865**

**(A)** Immunoblot analysis of 3T3 cells expressing *KRAS* G12A, G12R, or *HRAS* G12V, treated with increasing concentrations of BI-2865 (0–10 μM). Phospho-MEK1/2 (p-MEK1/2) was used to assess downstream MAPK signaling activity. Cleaved caspase-3 was assessed as a marker of apoptosis. GAPDH served as a loading control. **(B)** Immunoblot analysis of 3T3 cells expressing various rare or predicted oncogenic *KRAS* variants, including insertion/deletion mutations, with or without 1 μM BI-2865 treatment. Expression levels of KRAS, MEK1/2, and p-MEK1/2 were evaluated. GAPDH was used as a loading control.
